# Burnout in the Pharmaceutical Activity: The Impact of COVID-19

**DOI:** 10.3389/fpsyt.2021.771462

**Published:** 2022-01-20

**Authors:** Pedro Machado dos Santos, Claudia Ribeiro da Silva, Diana Costa, Carla Torre

**Affiliations:** ^1^CINTESIS - Center for Health Technology and Services Research, Porto, Portugal; ^2^Instituto de Ciências Biomédicas Abel Salazar da Universidade do Porto, Porto, Portugal; ^3^Escola Superior de Saúde do Alcoitão, Lisbon, Portugal; ^4^Ordem dos Farmacêuticos, Lisboa, Portugal; ^5^Faculdade de Farmácia da Universidade de Lisboa, Lisbon, Portugal; ^6^Laboratory of Systems Integration Pharmacology, Clinical and Regulatory Science, Research Institute for Medicines (iMED.ULisboa), Lisbon, Portugal

**Keywords:** COVID-19, maslach burnout inventory, mental health, pharmacists, pharmacy, burnout profiles

## Abstract

**Importance:**

Pharmacists are among the healthcare professionals involved in the response to the COVID-19 pandemic, maintaining essential services. In the context of restrictions and reorganization of human resources, as a result of policies recently applied to the health sector, following international guidelines, and given the scarcity of data on burnout in pharmaceutical activity, it was considered highly relevant to promote a nationwide survey aiming to collect more complete evidence on the burnout syndrome and to understand how pharmacists have viewed their work and the people they worked closely with.

**Objectives:**

The study aimed to assess the occurrence of pharmacist burnout and determine outcomes for each of its dimensions; identify potentially associated characteristics; and determine profiles and critical limits.

**Design:**

A cross-sectional observational study conducted by a multidisciplinary panel from the Portuguese Pharmaceutical Society (PPS). Professionals from community and hospital pharmacies who were at the forefront of the COVID-19 response were involved in (i) confirming the need and pertinence for conducting this research, (ii) identifying the main factors leading to pharmaceutical emotional distress (“burnout”), and (iii) disseminating the survey. The questionnaire was designed for digital voluntary, confidential, and anonymous participation and divided into four segments of data collection: (i) demographics, (ii) employment and workplace characterization, (iii) pandemic impact on labor activity, and (iv) burnout assessment (as described ahead).

**Setting:**

An electronic survey was addressed to all PPS members, and an account was created solely to manage the questionnaire data for the research team. The web-based and user-friendly platform Google Forms supported the data capture and provided an intuitive interface for validated data entry.

**Participants:**

In a population of 15,565 pharmacists (members of the PPS), the minimum recommended sample size (Epi Info software), with a 5% margin of error and a 99.9% confidence interval, should be 1,012 individuals. A total of 1,362 pharmacists participated in the study. Of these, 91.4% (*n* = 1,246) were involved in direct patient care activity and 7.7% (*n* = 106) in non-direct patient care activity.

## Highlights

- This research identified five different profiles on the continuum between “burnout” (the most negative experience) to “engagement” (the most positive), based on a person-centered analysis of pharmacists' burnout scores.
- Each profile has distinct relationships with various work–life factors, which suggests that each profile reflects a different work–life crisis that would require a unique intervention strategy.
- Having more confidence as a healthcare professional, on-the-job training, and direct patient care interactions may account as a buffering effect on burnout during the COVID-19 pandemic.

## Key Points

**Question:** What are the outcomes, profiles, and critical thresholds for each of the three burnout dimensions, as well as potentially associated characteristics, in the pharmaceutical activity during the COVID-19 pandemic?

**Findings:** A new profile-based approach provides an understanding of the burnout experience and distinct relationships with work–life factors and suggests that each profile reflects a different work–life crisis that would require a unique intervention strategy. Data indicate that pharmacists who have been involved in direct care activities are at the highest risk of burnout.

**Meaning:** Most pharmacists were able to avoid depersonalization and cynicism and thereby the burnout syndrome.

## Strengths and Limitations of this Study

**Strengths**

This was the first study to look at the impact of COVID-19 on pharmacists across Portugal with a large sample size across all pharmacy sectors.

This new focus on burnout profiles points to some new paths for future research and intervention on this important global problem.

**Limitation**

Some non-direct patient care activity groups may have been disproportionately represented with larger numbers of responses from some contexts, for example, community and hospital pharmacists compared with regulatory affairs or pharmaceutical distribution.

Of the participants, 85.5% were women, potentially skewing results due to sample bias.

The present results, indicating very positive developmental trends for work engagement and burnout, may not be applied to all occupational status groups.

## Introduction

Burnout syndrome has been defined as a psychological disorder caused by excessive stress due to overload or overwork. In other words, it is a complex response to prolonged or chronic job stress (1, 2).

Despite appearing in the 10th revision of the World Health Organization (WHO) International Classification of Diseases (ICD)—chapter XXI: “factors influencing health status or contact with health services” — “burnout” was only approved by the WHO as an “occupational phenomenon” in May 2019 and will be officialized for the first time in the 11th revision of the ICD (ICD-11), scheduled for the year 2022 (2, 3).

In the ICD-11 proposal, this syndrome “results from chronic stress in the workplace that has not been well-managed.” It refers specifically to phenomena in the professional context and should not be applied to describe experiences in other areas of life. In describing “burnout,” the WHO contemplates the presence of three dimensions: (i) feelings of energy depletion or exhaustion; (ii) increased mental distance from one's job or feelings of negativism or cynicism (CY) related to one's job; and (iii) reduced professional efficacy (PE) (2).

Although it is possible to find many evidence on burnout in healthcare professionals, most studies are focused on nurses and physicians (4, 5) and less on the pharmacist profession (6). According to Hall and colleagues, nurses had the greatest representation in the systematic review of 27 studies, followed by physicians, whereas pharmacists were represented in only two studies (4).

Like other healthcare professionals, pharmacists face many of the same challenges other providers do and are also prone to burnout due to several common and specific professional factors. This is also considering a highly regulated profession, with a continued focus on improving safety, therapeutic outcomes, and patient quality of life (7).

Despite the lack of research specific for pharmacists, evidence emerged showing that these professionals also suffered from burnout symptoms.

Moreover, the world, the healthcare sector, and the pharmaceutical context in particular have undergone a huge change with a great influence on the delivery of pharmaceutical services. On March 11, 2020, the WHO declared the novel coronavirus disease 2019 (COVID-19) caused by the severe acute respiratory syndrome coronavirus 2 (SARS-CoV-2) a pandemic (8).

In the first 6 months of the pandemic, the World Health Organization (9) reported 15,581,009 confirmed cases including 635,173 deaths in 213 countries.

COVID-19 has severely affected the delivery of healthcare services worldwide and brought further pressures, causing additional stress and increased workload for healthcare workers and pharmacy teams.

As healthcare workers, pharmacists played a role in hindering the spread of coronavirus by increasing patient awareness, especially by advising them on precautionary measures and providing appropriate information, and they were sometimes the source to provide protection products such as surgical masks or alcohol-based hand rub solutions. Community pharmacists were one of the most accessible healthcare professionals during this public health crisis, but all professional sectors have been functioning in a delicate balance of supporting users and families, colleagues, loved ones, and themselves.

In the national context of restrictions and reorganization of human resources, as a result of policies recently applied to the health sector, following international guidelines, and given the scarcity of data on burnout in pharmaceutical activity (mostly reported in academic studies), it was considered highly relevant to promote a nationwide survey aiming to collect more complete evidence on burnout syndrome among pharmacists and to understand how they have viewed their work and the people they worked closely with over the past 3 months and, by comparison, in the months prior to the pandemic (i.e., normative periods).

Therefore, the primary objective of this study was to determine pharmacists' outcomes for each of the three burnout dimensions: emotional exhaustion (EE), depersonalization (DP)/CY, and personal accomplishment (PA)/PE. Secondary objectives included (i) an overall assessment of pharmacist burnout, (ii) identification of potential characteristics associated with burnout, and (iii) determination of profiles and critical limits (cutoff points) for each of the three burnout dimensions.

## Methods

### Study Design

The “Burnout Pharmaceutical Survey” was conducted by the Portuguese Pharmaceutical Society (PPS) with the support of the Center for Health Technology and Services Research (CINTESIS).

An advisory group of pharmacists, including academics and representatives of the PPS, was set up to co-design the study, to develop the survey questions, and to analyze the results. This multidisciplinary panel focused primarily on recent surveys about the impact of the COVID-19 pandemic on healthcare activity and previous healthcare professional burnout studies published in peer-reviewed journals.

The questionnaire was designed for digital voluntary, confidential, and anonymous participation. In total, 20 sections of multiple-choice questions were selected by the authors, favoring quicker and more spontaneous answers. Survey questions were divided into four segments of data collection: (i) demographics, (ii) employment and workplace characterization, (iii) pandemic impact on labor activity, and (iv) burnout assessment (as described ahead).

Consensual review and validation were performed using a panel of experts who assessed and compared the different questions, according to the main board issues, semantics, and idiomatic and conceptual equivalent of the items' contents. If determined as not applicable, then it was modified to be more applicable to either healthcare providers (pharmacist patient care services) or non-providers. If there was no consensus, the majority of five panel members ruled on any issue.

### Patient and Public Involvement

Two pharmacists were involved as research team members.

Professionals from community and hospital pharmacies who were at the forefront of the COVID-19 response were involved in (i) confirming the need and pertinence for conducting this research, (ii) identifying the main factors leading to pharmaceutical emotional distress (“burnout”), and (iii) disseminating the survey.

A summary of results will be co-produced with pharmacists and disseminated to pharmacy professionals through the PPS, International Pharmaceutical Society (FIP), and other representative organizations.

### Administration of Online Survey

Google Forms was used to set up the survey online and to assemble responses, since it automatically hosts the online questionnaire via a unique URL and has proven to be an efficient and cost-effective platform to administer questionnaires without sacrificing quality, security, and fidelity of data (10). This web-based and user-friendly platform was designed to support data capture for surveys and research studies, providing (i) an intuitive interface for validated data entry; (ii) automated export procedures for seamless data downloads to common statistical packages; and (iii) procedures for importing data from external sources. As recommended, an account was created solely to manage the questionnaire data for the research team.

A pretest was performed with a convenience sample of 20 pharmacists, who reported that there were no issues with the contents of the survey. The final version was uploaded online using the PPS website.

### Ethical Approval

The institutional review board of the PPS approved the project as exempt and without ethical reservations to its resolution, since (i) the procedures concerning the anonymity of the data and the information provided to the volunteers are foreseen and (ii) the nature of the elements to be collected does not carry the risk of revealing weaknesses unknown to the volunteer and thus triggering unpredictable reactions.

### Data Collection and Inclusion Criterion

The online survey was distributed via e-mail to all members of the PPS. To boost the potential for response, the questionnaire was disseminated through several organizations and networks including pharmaceutical sectorial and professional associations. It was also posted on social networks. The survey was cascaded by providing details of the study, soliciting participation in the survey, and inviting pharmacists to share it with their professional networks and colleagues.

Selecting the electronic link served as first consent to participate voluntarily in the study. Information on the front page also confirmed that the survey was strictly confidential and in accordance with the applicable data protection law. Nevertheless, before starting the survey, study participants confirmed through a mandatory selection box that this was the first time completing this survey (ensuring 100% consent rate and preventing multiple responses) and acknowledged (i) the ethical terms and (ii) the inclusion criterion as being a certified member of the PPS. No personal identifiers were linked to survey results to ensure privacy, and the data, used only for research purposes, were maintained by the research team on a password-protected link. Contact e-mail from PPS was included for additional information.

### Outcome Measures

As in other studies assessing healthcare provider burnout, demographic and workplace characteristics collected in the survey included age, gender, number of people living in the household, years of practice, number of hours and weekends worked in the past 3 months (pandemic period), and degree of confidence in providing pharmaceutical care to patients with COVID-19.

### Burnout Survey Instrument

Burnout was measured using the Maslach Burnout Inventory (MBI) (11), currently recognized as a gold-standard tool (reliable, valid, and easy to administer) and the leading instrument to assess burnout (12).

The Portuguese versions of the MBI—Human Services Survey (HSS) and General Services (GS)—were used in this study after authorization from the copyright holder of the instrument (13).

The 22 standardized questions from the MBI-HSS and the 15 questions from the MBI-GS reported the feelings at work, to be answered by means of a seven-point frequency scale ranging from 0 (never) to 6 (always). The instrument assesses the three major dimensions or constructs of burnout syndrome: EE, DP/CY, and lack of PA/PE, with each aspect measured by a separate scale. For EE and DP/CY subscales, high average values correspond to high levels of burnout symptoms. Low average values in the PA/PE subscale correspond to high levels of burnout symptoms. Although independent from the other two, PA/PE cannot be seen as an opposition (10).

In the literature, it is possible to find a wide range of score cutoffs for burnout based on MBI results (14).

Previously, the original authors of the study and manual proposed statistical methods and a simplified approach to stratify burnout based on each subset score (15). Using the MBI scale definition of burnout, a high EE score of ≥27, a high PD score of ≥14, and/or a low PA score of ≤37 were considered. These cutoffs have been used more frequently in the relative health professional literature (13).

Cutoff scores were published in the third edition of the MBI manual (15) but were not included when the fourth edition was written (16).

For a more rigorous outcome measures and aiming to do a better risk assessment, the most recent guidelines were followed (17). These contribute to identify different clinical profiles (Table 1), combining the results of the three subscales (EE, DP/CY, and PA/PE) and based on “cut-off points” adjusted to the population under study (16).

**Table 1:** Clinical profiles and standardized critical boundaries based on the results of the burnout assessment.

**Profile**	**Dimensions**	**Cutoffs**		**Description**	**Example**
		**MBI-HSS**	**MBI-GS**		
Engaged	EE	≤3.00	≤2.90	Emotional stability, empathy, and professional fulfillment.	Professional with the ability to regulate himself emotionally, to empathize with others, and with feelings of professional effectiveness.
	DP/CY	≤2.73	≤2.86		
	PA/PE	>4.66	>4.30		
Ineffective	EE	≤3.00	≤2.90	Low effectiveness/achievement rate (no exhaustion or DP/CY problems).	Professional that reflects a loss of confidence in one's abilities, perhaps as a result of seemingly routine work or an environment that offers little recognition for a job well-done.
	DP/CY	>2.73	>2.86		
	PA/PE	**≤4.66**	**≤4.30**		
Overextended	EE	**>3.00**	**>2.90**	More acute form related to work overload (exhaustion).	Professional who is dedicated to his work and has a strong sense of efficiency, but who feels exhausted due to long working hours—he is accomplished and involved, but very tired.
	DP/CY	≤2.73	≤2.86		
	PA/PE	>4.66	>4.30		
Disengaged	EE	≤3.00	≤2.90	Disconnection with the organization, its members, culture or values—high DP/CY score (not overwhelmed)	Professional who demonstrates problematic relationships with co-workers and/or organizational values.
	DP/CY	**>2.73**	**>2.86**		
	PA/PE	>4.66	>4.30		
Burnout	EE	**>3.00**	**>2.90**	Feeling of exhaustion, increased mental distance/feelings of work-related negativism or CY, and a sense of lack of PE/PA.	A state of EE, with disbelief in the usefulness of the job one does and low achievement, with a decrease in effectiveness due to lack of commitment, which can lead to leaving the profession.
	DP/CY	**>2.73**	**>2.86**		
	PA/PE	**≤4.66**	**≤4.30**		

*MBI-HSS, Maslach Burnout Inventory—Human Services Survey; MBI-GS, Maslach Burnout Inventory—General Services; EE, Emotional exhaustion; DP, Depersonalization; CY, Cynicism; PA, Personal accomplishment; PE, Professional efficacy*.

*The bold values are the altered values, in contrast to normative condition: 93Disengaged94 (characterized by high DP/CY only), 93Overextended" (high EE only), and 93Ineffective94 (low PA/PE only)*.

The results were analyzed separately, taking into account the pharmaceutical activity: direct patient care and non-direct patient care. The former, including the professional areas of (i) community pharmacy, (ii) hospital pharmacy, and (iii) laboratory medicine and/or human genetics, was assessed with MBI-HSS. The latter was composed of (iv) regulatory affairs, (v) pharmaceutical distribution, (vi) academia, and (vii) pharmaceutical industry and scientific research were assessed with the MBI-GS.

### Statistical Analysis Plan

Data were exported to SPSS statistical software for analysis.

The Epi Info software, developed by the Centers for Disease Control and Prevention, U.S. Department of Health and Human Services (18), allowed us to ascertain that in a population of 15,565 pharmacists (active members of the PPS), the minimum recommended sample size, with a 5% margin of error and a 99.9% confidence interval, should be 1,012 individuals.

The same formulas suggested by Leiter and Maslach (17) were used to determine the critical limits for each of the three dimensions of the MBI. These calculations were performed for the sample of subjects in direct patient care activity using the MBI-HSS scale (22 items) and for the sample of subjects who were not in care activity using the MBI-GS scale (16 items). In their study, the authors defined profiles using standardized (*z*) values for the sample (17). Specifically, the critical threshold was defined as follows: (i) for EE at z = M + (SD × 0.5); (ii) for DP/CY at z = M + (SD × 1.25); and (ii) for PA at z = M + (SD × 0.10).

These critical thresholds thus vary with context and depend on population norms for the group. Therefore, the categorization of an individual's profile may slightly differ according to the population used to calculate the critical threshold.

Based on the critical limits, it was possible to perform a frequency analysis to determine in both samples how many subjects are below or above these limits in each of the three dimensions and establish the different profiles by combining the results in these factors.

In order to understand which variables may be related to burnout, a logistic regression model was used, since the dependent variable of burnout is dichotomous (Yes/No). The independent variables used were length of activity, number of working hours in the last 3 months, number of weekends worked in the last 3 months, degree of confidence to provide pharmaceutical care to patients with COVID-19, training in the COVID-19 area, living alone, and gender. Following the same rationale, three more logistic regressions were performed for each of the dichotomized MBI dimensions, using the same independent variables, to determine which ones might be related to high EE and DP/CY and low PA/PE.

The chi-square test was also used to relate the variable training in the COVID-19 area (trained vs. untrained) with the confidence in caring for patients with this disease (not at all or not very confident vs. very or very confident).

## Results

A total of 1,362 pharmacists participated in the study. Of these, 91.4% (*n* = 1,246) were involved in direct patient care activity and 7.7% (*n* = 106) in non-direct patient care activity.

Most respondents were women (85.5%), worked in Lisbon (29.8%), were full time (96%), were in the context of community pharmacy (77%), and had the possibility of receiving overtime (51.7%). The most represented group has been working for <5 years (23.2%), is between 31 and 40 years old (29%), is married/cohabiting (57.8%), lives with another person (28.3%), has no children under 12 (62.5%), and were not older adults over 65 (83.8%). While, in 2019, they had worked between 31 and 40 h/week (54.3%) and an average of 2 weekends per month (41.6%), in the first 3 months of the COVID-19 pandemic (2020), it changed to working between 41 and 50 h/week (39.8%). Although working 2 weekends a month remained the most common situation, there was an increase in the number of people who worked every weekend and a decrease in those who did not work on weekends.

The main sources of information to support the provision of care to COVID-19 patients were the health authorities (88.8%), the PPS (64.3%), and sector associations (44.4%).

Also, 41.1% of pharmacists were not confident in providing care to COVID-19 patients and were most concerned (i) about infecting their family (88.5%), (ii) about becoming infected while practicing (65.1%), and (iii) about the well-being of their family members (64.7%). Almost half of the respondents were concerned about mental health (41.5%), well-being of their colleagues (41.3%), and professional burnout (41.1%).

Professional activity was mostly influenced by worry about the future (89.6%), workload (82.9%), incomplete information and uncertainty (66.8%), and scarcity of resources (58.9%). Concern about the future (76.8%), workload (72.7%), patient volume (62.2%), incomplete information and uncertainty (62.0%), scarcity of resources (61.1%), and loss of control over the activity (54.1%) were the main influencing factors on work-related feelings.

In our study, we found that the most affected dimension among pharmacists in care activity was PA (50.1%) followed by EE (35.8%) and DP (12%), regardless of having changes in isolation or associated with another dimension. In the sample of subjects in non-care activity, the most affected dimension was PE (41.5%), followed by EE (33%) and CY (19.8%).

### Normative Data

In the present study, the same formulas suggested by Leiter and Maslach (17) were used to determine the critical thresholds and gauge the different profiles. These calculations were performed for the sample of subjects in care (*N* = 1,246) using the MBI-HSS scale (22 items) and for the sample of subjects who were not in care (*N* = 106) using the MBI-GS scale (16 items).

The critical boundaries found in this study for the different dimensions of the MBI-HSS and MBI-GS were respectively, EE ≥ 3.76 and EE ≥ 3.83; DP ≥ 2.83 and CY ≥ 4.11; and PA ≤ 3.56 and PE ≤ 4.99 (Table 2).

**Table 2:** MBI-HSS and MBI-GS scores to determine burnout.

**Scale**	***Burnout*** **dimensions**	***N***	**Mean**	**Std. deviation**	**Ex + SD × 0.5**	**Dep + SD × 1.25**	**Real + SD × 0.1**
MBI-HSS	EE	1,246	2.97	1.59	3.76		
	DP	1,246	1.3	1.23		2.83	
	PA	1,246	3.46	0.98			3.56
MBI-GS	EE	106	2.91	1.84	3.83		
	CY	106	2.01	1.68		4.11	
	PE	106	4.89	0.98			4.99

*MBI-HSS, Maslach Burnout Inventory—Human Services Survey; MBI-GS, Maslach Burnout Inventory—General Services; EE, Emotional exhaustion; DP, Depersonalization; CY, Cynicism; PA, Personal accomplishment; PE, Professional efficacy*.

**Figure 1:**
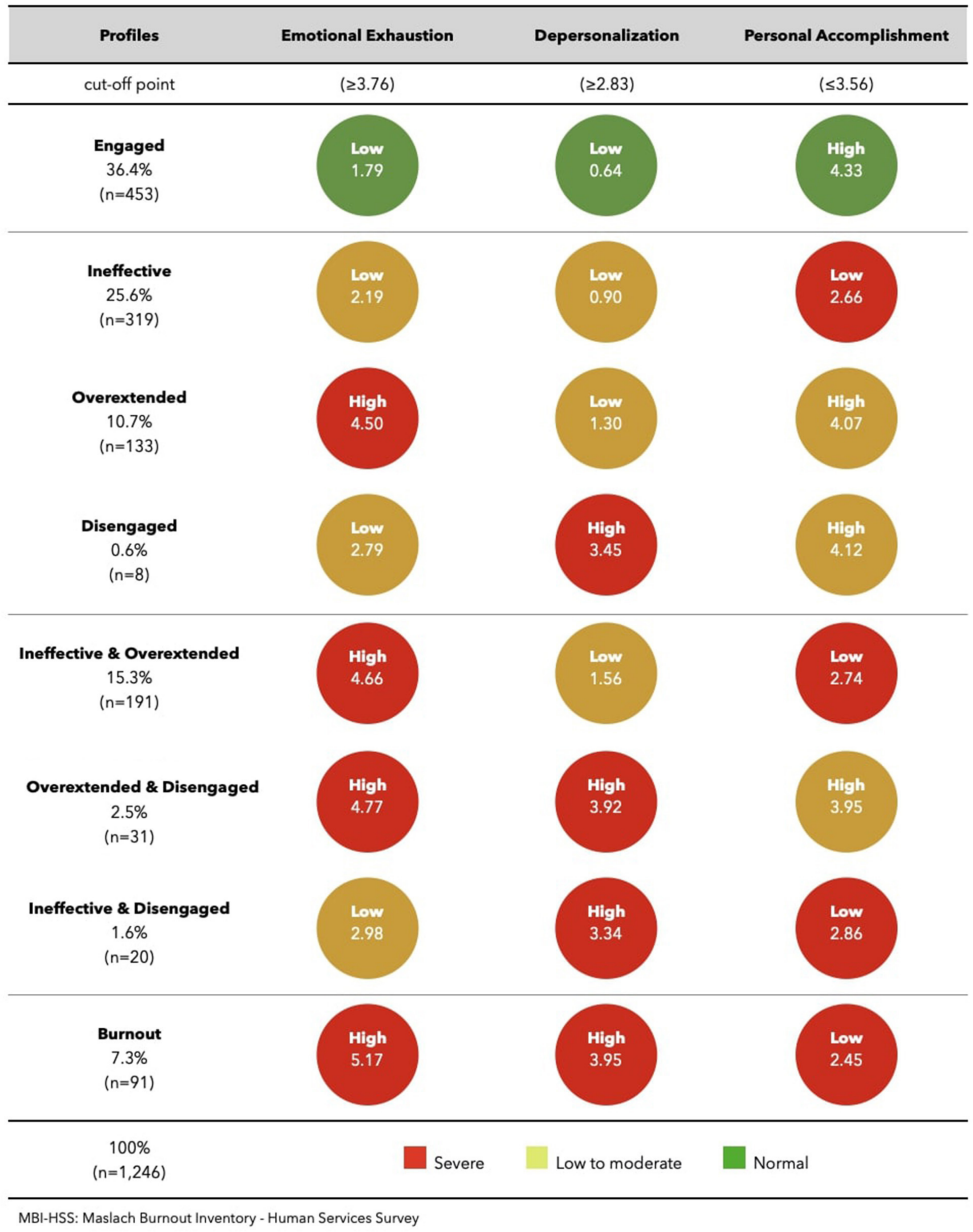
Pattern of MBI-HSS subscales across profiles: pharmacists involved in direct patient care activity.

In the sample of subjects who were involved in direct care activities, we found a total of 7.3% of subjects in burnout (with the three dimensions of the MBI-HSS affected). Without any affected dimension of the MBI, a total of 36.4% of subjects (“engaged”) were observed. With one dimension affected and at risk of burnout, there were 37.2% of subjects, and with two dimensions affected and at high risk of burnout, 19.4%. The most frequent profiles observed in this sample were the “engaged” profile (good results in the three dimensions) followed by the “ineffective” profile (25.6%), “overextended and ineffective” (15.3%) and “overextended” (10.7%) (Table 3). When observing the altered dimensions, it was found that the most affected, regardless of having alterations isolated or associated with another dimension, is PA (50.1%), followed by EE (35.8%) and lastly DP (12%) (Table 3 and Figure 1).

**Table 3:** Distribution of participants by clinical profile and burnout risk.

**Clinical profile**		**Direct patient care**		**Non-direct patient care**	
		**MBI-HSS** **(***n*** = 1,246)**		**MBI-GS** **(***n*** = 106)**	
		**RP**	**No. (%)**	**RP**	**No. (%)**
**Engaged** (normal)	low EE + low DP/CY + high PA/PE	1	453 (36.4)	1	48 (45.3)
Ineffective	low PA/PE	2	319 (25.6)	2	20 (18.9)
Overextended	high EE	4	133 (10.7)	4	9 (8.5)
Disengaged	high DP/CY	8	8 (0.6)	7	2 (1.9)
***Low to moderate risk***			460 (36.9)		31 (29.3)
Ineffective and overextended	low PA/PE + high EE	3	191 (15.3)	5	8 (7.5)
Overextended and disengaged	high EE + high DP/CY	6	31 (2.5)	6	3 (2.8)
Ineffective and disengaged	low PA/PE + high DP/CY	7	20 (1.6)	8	1 (0.9)
***High risk***			242 (19.4)		12 (11.2)
***Burnout***	high EE + high DP/CY + low PA/PE	**5**	**91 (7.3)**	**3**	**15 (14.2)**

*MBI-HSS, Maslach Burnout Inventory—Human Services Survey; MBI-GS, Maslach Burnout Inventory—General Services; EE, Emotional exhaustion; DP, Depersonalization; CY, Cynicism; PA, Personal accomplishment; PE, Professional efficacy; RP, Relative position (prevalence in sample)*.

**Figure 2:**
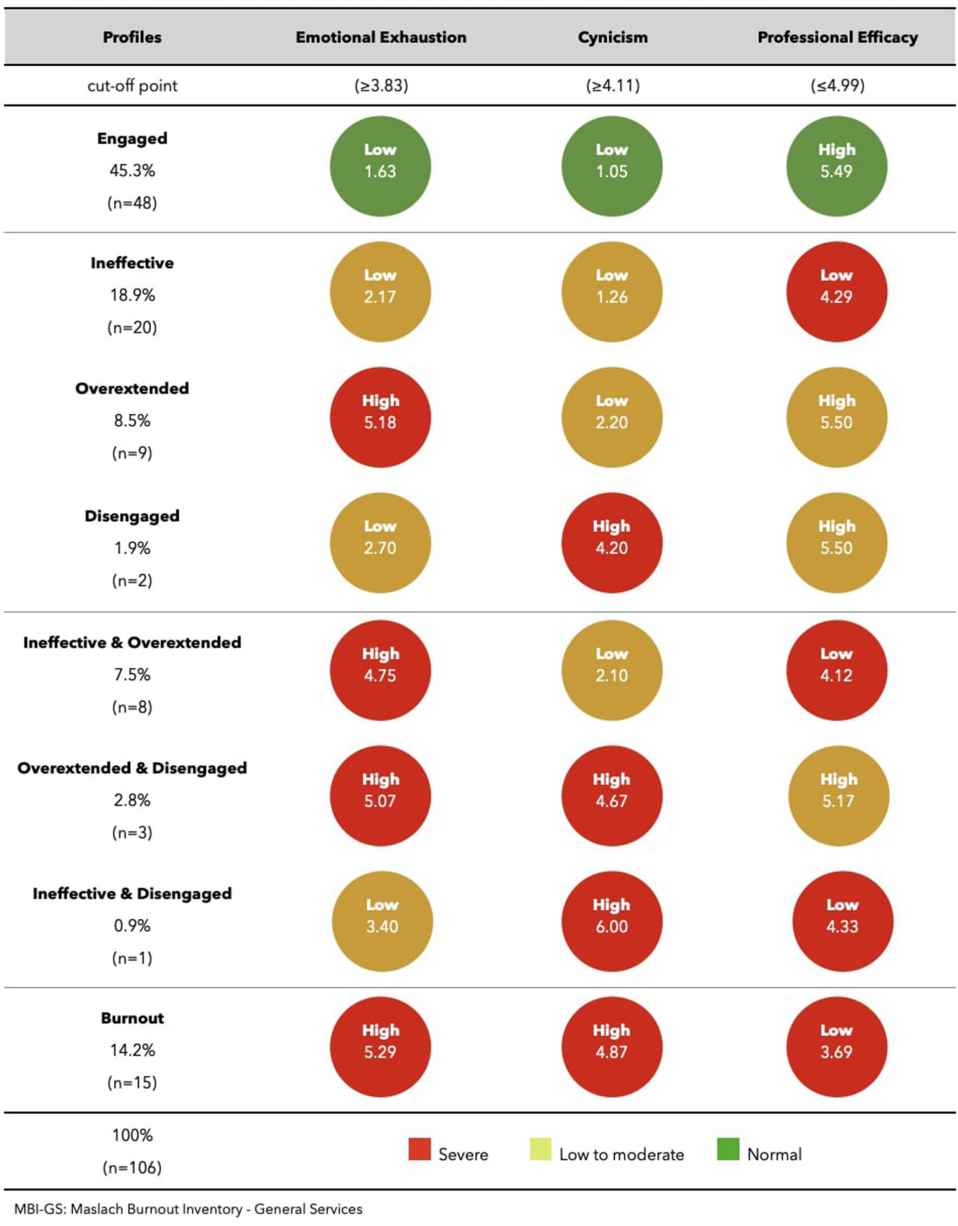
Pattern of MBI-GS subscales across profiles: pharmacists with non-direct patient care activity.

Among the sample of pharmacists who were not involved in direct care activities, we found 14.2% in a situation of “burnout” (with the three dimensions of the MBI-HSS affected), 11.2% with high risk of burnout (two dimensions affected), 29.3% at risk of burnout (one dimension affected), and 45.3% involved/engaged (no dimension affected). As for the sample profiles, we observe that the most frequent is the “engagement” (45.3%) (good results in the three dimensions), followed by the “ineffective” profile (18.9%) and “burnout” (14.2%) (Table 3 and Figure 2). With respect to the analysis of the altered dimensions, we see that the most affected is PE (41.5%) regardless of having alterations alone or associated with another dimension, followed by EE (33%) and lastly CY (19.8%).

Figures 1, 2 show the means of the three burnout dimensions for each profile, in the two main areas of activity: direct patient care activity and non-direct patient care activity.

### Dependent Variable Burnout

The omnibus test [G2(7) = 74.868, *p* = 0.000] revealed that there was at least one independent variable in the model with predictive power over the dependent variable burnout. The Hosmer and Lemeshow test revealed that the model is fitted [X^2^(8)_HL_ = 13.595, *p* = 0.093]. Through Wald's test, we can see that the variables, length of activity [X^2^(1) = 11.044, *p* = 0.001)] degree of confidence to provide pharmaceutical care to patients with COVID-19 [X^2^(1) = 26.739, *p* = 0.000], living alone [X^2^(1) = 11.994, *p* = 0.001], and gender [X^2^(1) = 14.320, *p* = 0.000], showed a statistically significant result on the logit of the probability of having burnout.

The probability of having burnout was (i) higher in those with shorter experience (1–5 years: 11.8; 6–10 years: 9.3; 11–15 years: 8.7; 16–20 years: 4.5; 21–25 years: 4.8; 26–30 years: 1.1% burnout; and 30 years or more: 2.2%); (ii) higher in those who lack confidence in providing care to patients with COVID-19 (not at all confident: 20%; not very confident: 7.6%; fairly confident: 5.1%; and very confident: 0.9%); (iii) higher in males (female: 6.5; male: 12.3%); and (iv) higher in those who live alone (does not live alone: 6.3% of burnout; living alone: 14.8%).

The three logistic regressions performed had omnibus test results that revealed the existence of at least one independent variable in the model with predictive power over the dependent variables: EE, DP, and (low) PA. The Hosmer and Lemeshow test revealed that the model is fitted in all three regressions (Table 4). The results for each of the dependent variables are presented below.

**Table 4:** Variables with predictive power over MBI-HSS scores (Logistic regression).

	**Independent variables**	**B**	**SE**	**Wald**	**Exp(B)**	**95% CI for EXP(B)**	
						**Lower**	**Upper**
	How long (in years) have you been working as a pharmacist?	−0.248	0.075	11.044^***^	0.780	0.674	0.903
DV: ***Burnout***	On average, how many hours per week have you worked in the past 3 months?	−0.059	0.126	0.220	0.943	0.736	1.207
	How many weekends have you worked in the past 3 months?	0.094	0.063	2.232	1.099	0.971	1.243
Omnibus: *X*^2^ = 74.868 *p* = 0.000	How would you describe your degree of confidence in providing pharmaceutical care to patients with COVID-19?	−0.808	0.156	26.739^***^	0.446	0.328	0.605
Hosmer and Lemeshow:	Have you completed any COVID-19-related training?	0.232	0.244	0.904	1.261	0.782	2.033
X^2^ = 13.595	Living alone	−0.997	0.288	11.994^***^	0.369	0.210	0.649
*p* = 0.093	Male	1.072	0.283	14.320^***^	2.920	1.676	5.086
	Constant	0.285	0.588	0.235	1.330		
	How long (in years) have you been working as a pharmacist?	−0.049	0.035	1.968	0.952	0.888	1.020
DV: ***Emotional exhaustion***	On average, how many hours per week have you worked in the past 3 months?	0.140	0.066	4.528^*^	1.150	1.011	1.308
	How many weekends have you worked in the past 3 months?	0.136	0.033	16,704^***^	1.145	1.073	1.222
Omnibus: X^2^ = 58.395 *p* = 0.000	How would you describe your degree of confidence in providing pharmaceutical care to patients with COVID-19?	−0.306	0.080	14.618^***^	0.737	0.630	0.862
Hosmer and Lemeshow:	Have you completed any COVID-19-related training?	−0.058	0.128	0.206	0.943	0.733	1.213
X^2^ = 10.826	Living alone	−0.630	0.194	10.560^***^	0.532	0.364	0.779
*p* = 0.212	Male	0.319	0.177	3.245	1.376	0.972	1.948
	Constant	0.022	0.337	0.004	1.022		
	How long (in years) have you been working as a pharmacist?	−0.176	0.055	10.233^***^	0.838	0.752	0.934
DV: **Despersonalization**	On average, how many hours per week have you worked in the past 3 months?	−0.034	0.098	0.124	0.966	0.798	1.170
	How many weekends have you worked in the past 3 months?	0.045	0.049	0.833	1.046	0.950	1.151
Omnibus: X^2^ = 64.568 *p* = 0.000	How would you describe your degree of confidence in providing pharmaceutical care to patients with COVID-19?	−0.543	0.119	20.673^***^	0.581	0.460	0.734
Hosmer and Lemeshow:	Have you completed any COVID-19-related training?	0.015	0.188	0.006	1.015	0.702	1.468
*X*^2^ = 15.024	Living alone	−0.801	0.242	10.916^***^	0.449	0.279	0.722
*p* = 0.059	Male	0.807	0.232	12.103^***^	2.241	1.422	3.532
	Constant	0.292	0.471	0.383	1.339		
	How long (in years) have you been working as a pharmacist?	−0.111	0.034	10.650^***^	0.895	0.837	0.957
DV: **Personal accomplishment**	On average, how many hours per week have you worked in the past 3 months?	0.015	0.064	0.052	1.015	0.895	1.150
	How many weekends have you worked in the past 3 months?	0.048	0.049	0.941	1.049	0.952	1.155
Omnibus: X^2^ = 69.351 *p* = 0.000	How would you describe your degree of confidence in providing pharmaceutical care to patients with COVID-19?	−0.439	0.078	31.562^***^	0.645	0.553	0.751
Hosmer and Lemeshow: X^2^ = 9.096 *p* = 0.334	Have you completed any COVID-19-related training?	0.339	0.124	7.507^**^	1.403	1.101	1.789
	Living alone	−0.164	0.195	0.707	0.849	0.580	1.243
	Male	0.427	0.176	5.885^*^	1.532	1.085	2.162
	Constant	1.542	0.338	20.839	4.673		

*MBI-HSS, Maslach Burnout Inventory—Human Services Survey; DV, Dependent variables; B, Estimated logit coefficient; SE, Standard error; Wald, Wald chi-square test; Exp(B), Exponentiation of the B coefficient (odds ratio); CI, Confidence interval*.

* *Statistically significant to p ≤ 0.05*.

** *Statistically significant to p ≤ 0.01*.

*** *Statistically significant to p ≤ 0.001*.

### Dependent Variable EE

Through Wald's test, we can see that the variables, number of hours worked in the last 3 months [X^2^(1) = 4.528, *p* = 0.03], number of weekends worked in the last 3 months [X^2^(1) = 16.704, *p* = 0.000], degree of confidence to provide pharmaceutical care to patients with COVID-19 [X^2^(1) = 14, 618, *p* = 0.000], and living alone [X^2^(1) = 10.560, *p* = 0.001], showed a statistically significant result on the logit of the probability of having EE.

The probability of having EE is (i) higher in those who worked more hours in the last 3 months (20–30 h: 23.5, 31–40 h: 35.9, 41–50 h: 34.4, and more than 50 h: 44.1%); (ii) higher in those who have worked more weekends in the last 3 months (no weekends: 28.3, 1–6 weekends: 28.9, 7–10 weekends: 44.2%, and all or almost all weekends: 45.4%); (iii) higher in those who lack confidence to provide care for patients with COVID-19 (not at all confident: 54.1%, not very confident: 36.5%, fairly confident: 30.6%, and very confident: 35.8%); and (iv) higher in those who live alone (does not live alone: 34.3%; living alone: 47.2%).

### Dependent Variable DP

Through Wald's test, we can observe that the variables, time in business [X^2^(1) = 10.233, *p* = 0.001], degree of confidence to provide pharmaceutical care to patients with COVID-19 [X^2^(1) = 20.673, *p* = 0.000], living alone [X^2^(1) = 10.916, *p* = 0.001], and gender [X^2^(1) = 12.103, *p* = 0.001], showed a statistically significant result on the logit of the probability of having DP.

The probability of DP is (i) higher in those who have shorter experience (1–5 years: 16, 6–10 years: 17.4, 11–15 years: 13.3, 16–20 years: 9, 21–25 years: 7.8, 26–30 years: 3.2, and 30 years or more: 6.7%); (ii) higher in those who are not confident to provide care to patients with COVID-19 (not at all confident: 23%, not very confident: 14.1%, fairly confident: 8.9%, and very confident: 5.5%); (iii) higher in males (female: 11.1, male: 17.9%); and (iv) higher in those who live alone (does not live alone: 11.0%; living alone: 20.4%).

### Dependent Variable (Low) PA

Through Wald's test, we can observe that the variables, time in business [X^2^(1) = 10.620, *p* = 0.001] degree of confidence to provide pharmaceutical care to patients with COVID-19 [X^2^(1) = 31.562, *p* = 0.000], completed training in COVID-19 area [X^2^(1) = 7.507, *p* = 0.006], and gender [X^2^(1) = 5.885, *p* = 0.015], showed a statistically significant result on the logit of the probability of having a low PA.

The probability of having a low PA is (i) higher in those with shorter experience (1–5 years: 57.3%, 6–10 years: 53% low achievement, 11–15 years: 53.8% low achievement, 16–20 years: 43% low achievement, 21–25 years: 42.2%, 26–30 years: 45.2%, 30 years or more: 44.4%); (ii) higher in those who lack confidence in providing care to patients with COVID-19 (not at all confident: 68.1%, not very confident: 53.7%, fairly confident: 45.3%, and very confident: 31.2%); (iii) higher in those who did not take COVID-19 training (no training: 54.8%; took training: 43.5%); and (iv) higher in males (male 55.3; female: 48.9%).

Since the variable degree of confidence in providing pharmaceutical care to patients with COVID-19 was shown in all regressions to be predictive of burnout and of the dimensions involved in burnout, we investigated whether those who had been trained in COVID-19 were the most confident in caring for patients with this disease.

The chi-square test revealed the existence of a significant association (*p* ≤ 0.001) between training in COVID-19 and confidence to provide care to patients with this disease: in the group of pharmacists who have taken training, there was a higher percentage with quite or a lot of confidence to provide pharmaceutical care to patients with COVID-19 (50.5%), while this percentage is significantly lower (only 38.7%) in the group that has not taken training.

## Discussion

Our study, with 1,362 participants, far exceeded the recommended minimum sample size of 1,012 subjects and represents one of the largest in the scope of burnout studies among pharmacists, at a global level.

According to a systematic review on pharmacist burnout that addressed studies about US pharmacists practicing in any setting, through February 13, 2019, the largest sample was obtained in a study published in 1990 with 1,258 professionals also working in multiple pharmacy-practicing settings (19).

So far, to the best of our knowledge, it is the most representative sample regarding studies conducted with members enrolled in health professional associations in Portugal—e.g., medical society and nurses society (5).

In the pandemic context, healthcare professionals who treated COVID-19 patients were found to have a 2.5 times higher risk of psychological distress than those who did not treat COVID-19 patients (20–22), and some authors have evidenced a relatively high prevalence of burnout symptoms among different healthcare professionals who had contact with COVID-19 patients. However, these recent studies have also shown great differences in their methodological approaches (e.g., samples, instruments, and measures).

At the first peak of the Italian COVID-19 pandemic, a large percentage of frontline healthcare professionals reported high scores in at least one of the MBI domains (23), and in Japan, a study conducted between April 6 and 19, 2020, showed that 7 (36.8%) of 19 pharmacists were experiencing burnout, following a primary criteria of high levels of exhaustion (>3.5) plus either high CY (>3.5) or low PE (<2.5), but adopted by the authors for the MBI-GS scale (24).

The SM-COVID-19 project, aimed at characterizing mental health and psychological well-being in the Portuguese context between May 21 and July 20, 2020, showed that among 815 health professionals, 32.1% had burnout, in this case associated with high scores of physical fatigue, EE and cognitive weariness on the Shirom–Melamed Burnout Measure (SMBM) (25). According to some studies, the healthcare professional groups most affected by burnout were those who were in regular contact with patients, who were treating patients with COVID-19, and who had increased work hours. Among the general population (i.e., excluding healthcare professionals), community pharmacy workers were the second most affected group (34%), just behind workers in institutions for older adults (39%) (26).

In the present work, we decided to use the leading instrument to assess burnout (MBI) and the most suitable versions for the professionals involved in direct and non-direct patient care activity (HSS and GS), since it allows comparison with most peer-reviewed articles, and it is currently recognized as a gold-standard tool that offers good psychometric guarantees.

The reliability of the MBI subscales was measured via internal consistency coefficient alpha (27). The Cronbach alphas obtained on the MBI-HSS and MBI-GS were 0.92 (EE), 0.75 (DP), and 0.81 (PA) and 0.93 (EE), 0.86 (CY), and 0.81 (PE), respectively.

These reliability coefficients show adequate internal consistency for each of the three MBI-HSS and MBI-GS scales and are in agreement with the wide range of samples, presented in the fourth edition of the MBI manual (16) and with a reliability generalization meta-analysis of coefficient alpha–of the 221 studies reviewed, 84 provided alpha coefficients—which, with respect to estimates of the untransformed means, shows sample size-weighted means and standard deviations of 0.87 (0.03), 0.74 (0.09), and 0.78 (0.05) for the EE, DP, and PA scales, respectively (28).

In addition to these guarantees, the new procedures presented in the latest manual proved also to be a more validated method for determining burnout, compared to the previous studies.

In practical terms, this research, based on a person-centered analysis of people's burnout scores conducted by Leiter and Maslach (17), also allowed identification of five different profiles on the continuum between “burnout” (the most negative experience) to “engagement” (the most positive) (Table 1). However and although we started attending the same three intermediate profiles— “disengaged” (characterized by high DP/CY only), “overextended” (high EE only), and “ineffective” (low PA/PE only)–we think that the seven-class model fit best to identify multiple person-centered forms of distress and profiles across the burnout (Table 3).

A person-centered approach considers the whole person, in contrast with an exclusive concern on single dimensions prioritized in variable-centered approaches, and can be useful for theory, research, and design of interventions.

The burnout profile is clearly more distressing than the “overextended,” but both have issues to be addressed. In the middle, we find a “disengaged” profile more negative than the “overextended” and closer to the “burnout” profile, which argues against the use of exhaustion alone as a proxy for burnout.

Standardized (*z*) values used to calculate profiles with the critical thresholds among pharmacists confirmed that these critical boundaries vary across the context and are dependent on the population norms for the group. In this study, it is possible to advance a new pharmacist's profile categorization, based on the population used in the critical boundary calculation (direct patient care and non-direct patient care) and slightly different from those proposed by the authors (13).

Surprisingly, professionals who were involved in direct care activities, represented mainly by community pharmacists (77%), had a higher risk of burnout (19.4%) but effectively less burnout (with all three dimensions affected) compared to pharmacists who were not involved in direct care activities (7.3 and 14.2%, respectively).

The proposed new approach, which we follow here, naturally makes this study less one-to-one comparable to previous studies (where historically only one subgroup was considered necessary for burnout) but will better compare with future studies done using burnout profiles. However, it becomes possible to draw some considerations with respect to studies conducted with pharmacists by taking scores obtained on the MBI subscales.

The prevalence results are in line with an investigation conducted in the United States with pharmacists in the healthcare system (29), where 53.2% of the study participants (*n* = 177) showed scores indicating a high degree of burnout on at least one dimension and 8.5% respondents (*n* = 28) showed scores indicating burnout on all three subscales of the MBI-HSS (high EE, high DP, and reduced PA). According to the authors, this burnout rate was similar to published findings for physicians and nurses.

Regarding the associations between burnout and primary work setting, our results does not appear to support previous studies. An earlier American research revealed that those pharmacists working primarily in community chain store settings reported higher levels of burnout than those working in hospital or institutional pharmacies, independent community pharmacies, academia, and home healthcare (30). An English study found that community pharmacists had significantly higher levels of workplace stress than other healthcare professionals (31). Most recently, a cross-sectional study also showed a high prevalence of psychological distress, work-related burnout, and compassion fatigue among hospital pharmacists in Japan (6).

However, the mentioned studies were conducted before the COVID-19 pandemic situation. In this case, pharmacists who were on the front line (in direct care activities), despite the high increase in professional demands, maintained their routines and became less isolated, compared to those who were not involved in direct care activities and had to drastically change their daily lives. Community pharmacists, for instance, who were one of the most accessible healthcare professionals during this public health crisis, and here most represented (77%), were always in contact with the public, reinforcing human relationships that avoid DP and CY and therefore burnout syndrome.

Nevertheless, our study results are framed in an early 2020 systematic review of burnout in pharmacists that revealed an overall estimate of the prevalence of burnout based on individual MBI subscales that ranged from 8 to 53% (32). The prevalence of burnout reported in six articles (40%), also using specific scores to define MBI measures of high EE and low PA, showed a wider range and lower than previously reported by individual studies, which cited overall burnout ranging from 52 to 61% using a composite of one or more MBI subscales.

In contrast to this systematic review, the proportion of pharmacists (involved in care and non-care activity) with MBI subscale scores consistent with burnout was greater for low PA (49.8 and 41.5%) than for high EE (35.8 and 33%). Yet the percentage of subjects with high DP (12 and 19.8%) was always lower in the different studies.

The already-mentioned meta-analysis showed different proportions in both groups of nine and six studies reviewed, but still greater for high EE (41 and 37%) than for low PA (32 and 33%). The proportion of pharmacists who met authors' definition of high DP was always lower (20 and 19%) (20).

Our results are not in agreement with specific burnout studies among pharmacists conducted before the pandemic period, such as the research with hospital pharmacists in Romania that identified a moderate level of EE, a low level for DP, and an average level of burnout for PA (33). In South Africa, researchers reported that 39% of pharmacists in community pharmacies, hospital, and executive positions exhibited moderate to high levels of EE (34), and in Turkey, community pharmacists revealed low levels of PA (35). And an American study revealed that pharmacists practicing on the front lines of insurance structures (*health maintenance organization*) showed also moderate levels of EE and DP (36).

However, the recent “latent profile analysis” (13, 17) using a new data modeling approach and multiple person-centered profiles in 1,766 healthcare employees, including a wide range of clinical, administrative, and support areas, has also highlighted “ineffective” (high inefficacy, moderate other) as the most frequent profile (31%), immediately after “engagement” (44%), an order of prevalence which is in line with our study: “ineffective” 25.6% (MBI-HSS) and 18.9% (MBI-GS) and “engagement” 36.4% (MBI-HSS) and 45.3% (MBI-GS).

In all above-mentioned studies, the hypotheses for the ineffective profile were less clear, perhaps because there has been less prior research on the efficacy dimension and because the existing findings do not show strong relationships between this dimension and the kind of workplace variables already assessed.

In our study, the difference on burnout subsets and an identifiable “high ineffective only” seems to be related to the current crisis contexts: the onset of the pandemic period and the role played by pharmacists in hindering the spread of coronavirus. While being less confident to provide care to patients with COVID-19 showed a statistically significant probability of having a low PA, high EE, and high DP and burnout as a whole, having not taken COVID-19 training has a statistically significant result only on low accomplishment.

Not only do these findings suggest that ineffectiveness and loss of confidence in one's abilities are a consequence of labor insecurity feelings, but most of all these also speak to the importance of improving confidence among health professionals in a crisis context.

Since pharmacists who had received training in COVID-19 were the most confident and showed a higher efficacy/outcome rate, it would appear clear that future contingency plans should include training for healthcare professionals, particularly those who have less work experience, creating a working environment that provides more recognition for a job well-done, as suggested by MBI authors (12).

Indeed, in our survey, the likelihood of having burnout, high PD, and low PA is higher in those with less work experience and probably less confident. This corroborates previous American research which revealed that pharmacists in their first 10 years of practice in all work settings had moderate levels of burnout (30).

More recent evidence has shown that the PA factor is significantly reduced in the “oldest” age category, but with an increasing length of practice, the average of EE factor also increases (37). The latter has not been yet confirmed in our study.

Workload in turn appears only as a predictor of high EE, with a great impact on those who worked during the 3-month period for more than 50 h/week (44.1%) and on those who worked almost every weekend (45.4%), with the latter having a greater predictive power of high EE (*p* ≤ 0.001). This not only underlines the importance of free time in an individual's well-being but also recalls the evidence that weekends are associated with high feelings of freedom and closeness—engaging in activities of your choice and time with close friends and family (38)—and that working extra-long hours and over weekends contributes to more depressive symptoms and worse mental health (39).

Outside a crisis, US researchers reported that two-thirds of pharmacists described their workload as high or excessively high, and 45% reported that their workload had negatively impacted their emotional and mental health (40).

In this recent and particular context, it is reasonable to expect that pharmacists living alone would have absorbed a greater workload and in a combined way led to a greater EE, DP, and burnout syndrome. Even if this single condition has not affected PA or on the contrary may improve this dimension, it is important to underline that isolation poses risks to mental health (41–43). Consequently, it would be important to consider measures that promote regular contact with significant people in order to prevent DP and CY and therefore burnout syndrome.

Until quite recently, it was expected that environmental factors of the modern healthcare workplace would arguably continue to contribute not only to EE but also to DP and to reduced PA in the foreseeable future (32). However, the knowledge acquired during the pandemic crisis may help professionals and decision-makers to break this trend and to clarify which individual and environmental weaknesses and strengths may contribute to explaining and coping better with burnout in pharmacists.

Despite the fact that pharmacists fall into virtually the same healthcare provider category, some pharmacy sectors have less information available (e.g., pharmaceutical industry). Our study follows the trend of representing community pharmacy more, in addition to relatively recent studies conducted among community pharmacists (31, 34, 35, 37, 44), but also at a pharmacy practice faculty (45) or among hospital pharmacists (6, 33). Nevertheless, overall, these reports reflect an emerging picture of burnout that has existed, and continues to exist, in the pharmacy profession.

The evidence had already shown that EE among nurses and physicians working in intensive care units was associated with an increased patient mortality (46), and a systematic analysis including providers from multiple healthcare professions showed an association between burnout and an increase of medical errors (4). A more recent meta-analysis of 47 studies on 42,473 physicians described physician burnout as an epidemic affecting healthcare delivery and patient safety (47). However, no pharmaceutical-specific papers were identified regarding the impact of burnout on job performance and patient safety.

It is also important to note that the period covered by COVID-19 is not considered a normal work situation. During the study period, Portugal was witnessing the worst outbreaks compared with neighboring countries in southern Europe (e.g., Spain and Italy) and was struggling with a rapidly worsening situation that reached 49,915 confirmed cases and some 1,716 deaths (48). In this context, it was plausible to assume that the pandemic could have exacerbated preexisting causes of burnout in pharmacists by adding its own stressors. However, not enough prior information was available to compare.

Therefore, it would be important to repeat the study after the pandemic, ideally collecting evidence about the association between pharmacist burnout and healthcare delivery and patient safety. Repeating the study at the international level (during and after the pandemic situation) may also help to verify the validity of the cutoff points, strengthen the findings, and compare them with future studies using burnout profiles.

## Conclusion

Burnout symptoms can affect pharmacists in all practice settings, yet most pharmacists do not meet the criteria for the individual dimensions of burnout. Inconsistencies on how this complex response to prolonged or chronic job stress has been measured in the literature limit conclusions and also recommendations for interventions.

This new profile-based approach provides an understanding of the burnout experience and distinct relationships with various work–life factors and suggests that each profile reflects a different work–life crisis that would require a unique intervention strategy. Therefore, it was extremely important to use this new methodology, determining the five MBI profiles for pharmacists and contributing to the identification of differentiated scores between healthcare professional groups.

Our data indicate that pharmacists who have been involved in direct care activities are at the highest risk of burnout compared with professionals practicing in other areas. However, they were able to avoid DP and CY and thereby burnout syndrome.

The syndrome is often associated with issues surrounding workload, professional experience, or degree of confidence in providing pharmaceutical care.

Prevention strategies such as focusing on work–life balance, peer support, continued education, and self-care may be effective in reducing the risk of burnout among pharmacists. Individuals who demonstrate greater professional, psychological, and social frailty, such as lack of experience, anxiety, depression, or social isolation, require greater mental health attention and support.

Unfortunately, burnout is not a new challenge in many different settings and workplaces. A growing body of literature supports the notion that burnout symptoms are prevalent among healthcare providers and yet associated with negative outcomes, such as suboptimal patient care and professional inefficiencies.

It is therefore opportune to continue and deepen the knowledge about pharmacist mental health and its impact on people benefiting from pharmaceutical care and on co-workers, in order to allow better comparison between different professional groups and contribute to the adjustment of health policies.

In conclusion, we can state without hesitation that the pandemic of COVID-19 has had a huge impact on the delivery of pharmaceutical care.

All efforts, logistical procedures, and patient counseling were essential for maintaining high-quality care and reducing harm, especially among the most vulnerable groups. In order to plan, provide better support to the pharmaceutical sector, and prevent future crises, as well as to potentially adapt healthcare services to the cycles of SARS-CoV-2, interventions to promote psychological well-being of pharmacists would need to be developed.

## Data Availability Statement

The authors confirm that the data supporting the findings of this study are available within the article [and/or] its supplementary materials.

## Ethics Statement

Ethical review and approval was not required for the study on human participants in accordance with the local legislation and institutional requirements. The patients/participants provided their written informed consent to participate in this study.

## Author Contributions

PS, DC, and CT: conceived and planned the experiments and carried out the experiments. CS and PS: analyzed the data. PS, CS, DC, and CT: contributed to the interpretation of the results. PS: took the lead in writing the manuscript. All authors provided critical feedback and helped shape the research, analysis, and manuscript.

## Funding

This work was supported by the PPS.

## Conflict of Interest

The authors declare that the research was conducted in the absence of any commercial or financial relationships that could be construed as a potential conflict of interest.

## Publisher's Note

All claims expressed in this article are solely those of the authors and do not necessarily represent those of their affiliated organizations, or those of the publisher, the editors and the reviewers. Any product that may be evaluated in this article, or claim that may be made by its manufacturer, is not guaranteed or endorsed by the publisher.
